# Plight of Dentistry in Pakistan

**Published:** 2020

**Authors:** Amjad H Wyne

**Affiliations:** Professor Dr. Amjad H Wyne BDS, BSc, MDS, Dr. Med. Dent., FASDC, FADI Head, Pediatric Dentistry Department, CMH Medical College Institute of Dentistry, Lahore. Co-Editor, Pakistan Oral & Dental Journal President, Pakistan Academy of Pediatric Dentistry Email: ahwyne@gmail.com

This refers to your editorial “Plight of Dentistry in Pakistan”. Published in Pakistan Journal of Medical Sciences.^1^ A very good description of current situation and historical prospective about dental education and journalism. I have recently written two guest editorials one each in “Pakistan Oral & Dental Journal” and “Journal of Pakistan Dental Association”, the only two regularly published dental journals in the country. In fact “Pakistan Oral & dental Journal” is in its 40th year of regular publication without any interruption^2^.

The topic of my editorials was plight of pediatric dentistry in Pakistan. While this subject has been taught as a separate major subject since more than a century in rest of the world, it remains neglected here. While almost 40% of our population is below 14 years of age, practically no training is given at undergraduate level to our dental graduates on how to treat dental diseases in pediatric dental patients.

Thanks so much for writing on the deteriorating situation of dental education in Pakistan.
